# Coexpression of EGFR and CXCR4 Predicts Poor Prognosis in Resected Pancreatic Ductal Adenocarcinoma

**DOI:** 10.1371/journal.pone.0116803

**Published:** 2015-02-13

**Authors:** Huanwen Wu, Liang Zhu, Hui Zhang, Xiaohua Shi, Li Zhang, Wenze Wang, Huadan Xue, Zhiyong Liang

**Affiliations:** 1 Department of Pathology, Peking Union Medical College Hospital, Chinese Academy of Medical Science, Beijing, China; 2 Department of Radiology, Peking Union Medical College Hospital, Chinese Academy of Medical Science, Beijing, China; University of Verona, ITALY

## Abstract

**Background:**

Epidermal growth factor receptor (EGFR) is highly expressed in pancreatic ductal adenocarcinoma (PDAC) and is involved in tumorigenesis and development. However, EGFR expression alone has limited clinical and prognostic significance. Recently, the cross-talk between EGFR and G-protein-coupled chemokine receptor CXCR4 has become increasingly recognized.

**Methods:**

In the present study, immunohistochemical staining of EGFR and CXCR4 was performed on paraffin-embedded specimens from 131 patients with surgically resected PDAC. Subsequently, the associations between EGFR expression, CXCR4 expression, EGFR/CXCR4 coexpression and clinicopathologic factors were assessed, and survival analyses were performed.

**Results:**

In total, 64 (48.9%) patients expressed EGFR, 68 (51.9%) expressed CXCR4, and 33 (25.2%) coexpressed EGFR and CXCR4. No significant association between EGFR and CXCR4 expression was observed (*P* = 0.938). EGFR expression significantly correlated with tumor differentiation (*P* = 0.031), whereas CXCR4 expression significantly correlated with lymph node metastasis (*P* = 0.001). EGFR/CXCR4 coexpression was significantly associated with lymph node metastasis (*P* = 0.026), TNM stage (*P* = 0.048), and poor tumor differentiation (*P* = 0.004). By univariate survival analysis, both CXCR4 expression and EGFR/CXCR4 coexpression were significant prognostic factors for poor disease-free survival (DFS) and overall survival (OS). Moreover, EGFR/CXCR4 coexpression significantly increased the hazard ratio for both recurrence and death compared with EGFR or CXCR4 protein expression alone. Multivariate survival analysis demonstrated that EGFR/CXCR4 coexpression was an independent prognostic factor for DFS (HR = 2.33, *P*<0.001) and OS (HR = 2.48, *P* = 0.001).

**Conclusions:**

In conclusion, our data indicate that although EGFR expression alone has limited clinical and prognostic significance, EGFR/CXCR4 coexpression identified a subset of PDAC patients with more aggressive tumor characteristics and a significantly worse prognosis. Our results suggest a potentially important "cross-talk" between CXCR4 and EGFR intracellular pathways and indicate that the simultaneous inhibition of these pathways might be an attractive therapeutic strategy for PDAC.

## Introduction

Pancreatic ductal adenocarcinoma (PDAC) exhibits the poorest prognosis of all solid tumors, with a 5-year survival rate of less than 5% and a median survival of 6 months after diagnosis [1]. A better understanding of PDAC pathogenesis and the identification of new prognostic markers and therapeutic targets are urgently needed.

Recent translational studies have reported a large number of potentially relevant biomarkers in PDAC. Epidermal growth factor receptor (EGFR) is a member of the c-erbB membrane receptor family. The EGFR pathway has been found to play important roles in tumorigenesis and development, and EGFR overexpression has been observed in many epithelial cancers, including lung, breast, colon, and prostate cancers [2], [3]. Moreover, specific EGFR inhibition has been one of the key targets for cancer therapy [2]. PDAC also displays overexpression of EGFR. The association between EGFR expression and the clinical outcome in PDAC has long been studied, but the results have been controversial [4]. Another promising marker of PDAC is the CXC chemokine receptor 4 (CXCR4). As a G-protein-coupled chemokine receptor, CXCR4 exerts its biological effect by binding its sole known natural ligand CXCL12 (also known as SDF-1). CXCL12/CXCR4 is a critical signaling pathway in the regulation of leukocyte recruitment and embryo development and has recently been demonstrated to play important roles in cancer invasion, metastasis, angiogenesis, cancer cell-microenvironment interaction and chemoresistance [5], [6]. The CXCL12/CXCR4 pathway has also been reported to be involved in pancreatic development and PDAC pathogenesis [7], [8]. Moreover, high CXCR4 expression has been found to be an independent prognostic biomarker associated with lymph node metastases and distant metastatic recurrence in resected PDAC [9], [10].

Recently, the “cross-talk” between EGFR and CXCR4 signaling pathways has been observed in many solid malignancies, including non-small cell lung, breast, gastric and ovarian cancers [11–15], and the coexpression of EGFR and CXCR4 might define a new molecular subtype displaying a worse prognosis in non-small cell lung cancer and breast cancer [15], [16].

In the present study, we investigated the expression of EGFR and CXCR4 in resected PDAC tissues, analyzed the correlation between EGFR and CXCR4 expression, and first assessed the clinical and prognostic significance of EGFR/CXCR4 coexpression in PDAC.

## Materials and Methods

### Patients

PDAC patients who had undergone surgery with curative intent during the period between January 2006 and March 2014 were retrospectively reviewed from the surgical pathology files of Peking Union Medical College Hospital. All types of pancreatic resections were eligible. The exclusion criteria included preoperative chemotherapy (CT) or radiotherapy (RT), macroscopic incomplete resection (R2), or inadequate follow-up data. Patients who had a survival time of less than 30 days from the time of surgery were also excluded to eliminate the confounding factor of perioperative mortality. After a review of the clinical and pathologic data, a total of 131 patients with PDAC were included in our study. The institutional review board of Peking Union Medical College Hospital approved the study, and written informed consent was obtained.

### Clinicopathologic data

The medical records were thoroughly reviewed for age, gender, date of surgery, date of relapse, and date of death or date of last follow-up. The pathologic findings (tumor size, lymph node status, resection margins and tumor differentiation) were obtained from the pathologists’ original reports and were verified by experienced pathologists (HW and HZ). Patients were staged according to the tumor-node-metastasis (TNM) system.

### Immunohistochemical staining and scoring

Immunohistochemical staining was performed using the EnVision system (DAKO, Glostrup, Denmark). Briefly, serial 5-μm-thick sections were cut from formalin-fixed and paraffin-embedded tumor blocks, dewaxed in xylene, rehydrated through sequential changes of alcohol, and then antigen retrieved using 0.01 M citrate buffer, pH 6.0, at 90°C for 20 min. After the tissue sections were washed with phosphate-buffered saline (PBS), they were incubated with fresh 3% hydrogen peroxide for 20 min at room temperature. The sections were then blocked with 20% goat serum for 30 min and incubated with EGFR primary antibody (1:200 dilution; Abcam, Cambridge, UK) or CXCR4 primary antibody (1:50 dilution; Abcam) for 2 h. The sections were then incubated with a polymer HRP secondary antibody (DAKO). Immunostaining was finally developed with 3,3′-diaminobenzidine (DAB). Positive and negative controls were run as appropriate. The sections were assessed independently by three experienced pathologists (HW, HZ and XS) who were blinded to both the clinical and the pathology data. The scoring for each section was determined by consensus.

The expected staining pattern was cytoplasmic and/or membranous for both EGFR and CXCR4. Immunohistochemical staining for EGFR was scored as previously described [17], [18]: 0, no staining; 1+, weak staining; 2+, moderate staining; and 3+, strong staining. Samples with more than 10% of the tumor cells showing membranous staining and/or cytoplasmic staining at the 2+ and 3+ staining levels were considered to be EGFR positive. For CXCR4, immunoreactivity was assessed using the semi-quantitative immunoreactive score (IRS) [10]. The IRS was calculated by multiplying the staining intensity (0 = no staining; 1 = weak staining; 2 = moderate staining; and 3 = strong staining) by the percentage of positively stained cells (0 = 0%; 1 = 1–30%; 2 = 31–60%; and 3 = >60%). IRS scores >3 were considered CXCR4 positive.

### Statistical analysis

Disease-free survival (DFS) was defined as the interval from the date of surgery to the first documented local or distant recurrence or the last follow-up. The date of recurrence was defined as the date of the first subjective symptom indicating relapse or the date of documentation by diagnostic imaging techniques for recurrent disease. Overall survival (OS) was calculated from the date of surgery to death from any cause. For unknown deaths, patients were censored at the date of last follow-up. Patient follow-up time was calculated using the reverse Kaplan–Meier method. The χ² test was used to analyze the associations between categorical variables. Survival curves were calculated according to the Kaplan-Meier method and compared using the log-rank test. In the univariate survival analysis, we analyzed the influence of the following individual factors on survival: age, sex, tumor sites, lymph node metastasis, TNM stage, resection margins, tumor differentiation, EGFR expression, CXCR4 expression and EGFR/CXCR4 coexpression. A Cox proportional-hazards regression model was used to estimate hazard ratios and 95% confidence intervals (CIs) and to perform multivariate survival analysis using a forward variable selection procedure. Variables with significant or borderline significant values (*P*≤0.10) in a univariate analysis were included in the multivariate analysis. A Cox regression model was also used to investigate whether EGFR/CXCR4 coexpression significantly worsened outcome compared with either EGFR or CXCR4 expression alone. The level of significance was defined as *P*≤0.05 (two-tailed). All of the data analyses were performed using SPSS software for Windows, version 20 (SPSS Inc., Chicago, IL, USA).

## Results

### Patient characteristics and EGFR and CXCR4 expression

One hundred and thirty-one patients were included in our case series. The characteristics of the patients are summarized in Table 1; 75 men and 56 women with a median age of 61.0 years (range: 15–80 years) were included.

**Table 1:** Summary of baseline patient characteristics (n = 131).

Parameter	No. of patients (%)Number(%)
**Total**	131 (100.0)
**Age range (years)**	15–80
Median age (years)	61.0
Mean age (years)	59.9
<60	61 (46.6)
≥60	70 (53.4)
**Gender**	
Male	75 (57.3)
Female	56 (42.7)
**Tumor sites**	
Head	85 (64.9)
Body/tail	46(35.1)
**Tumor size**	
T1–2	59 (45.0)
T3–4	72 (55.0)
**Lymph node metastasis**	
No	63 (48.1)
Yes	68 (51.9)
**TNM stage**	
I-II	119 (90.8)
III	12 (9.2)
**Resection margins**	
Negative	100 (76.3)
Positive	31 (23.7)
**Tumor differentiation**	
Well/moderate	84 (64.1)
Poor	47 (35.9)

**Figure 1:**
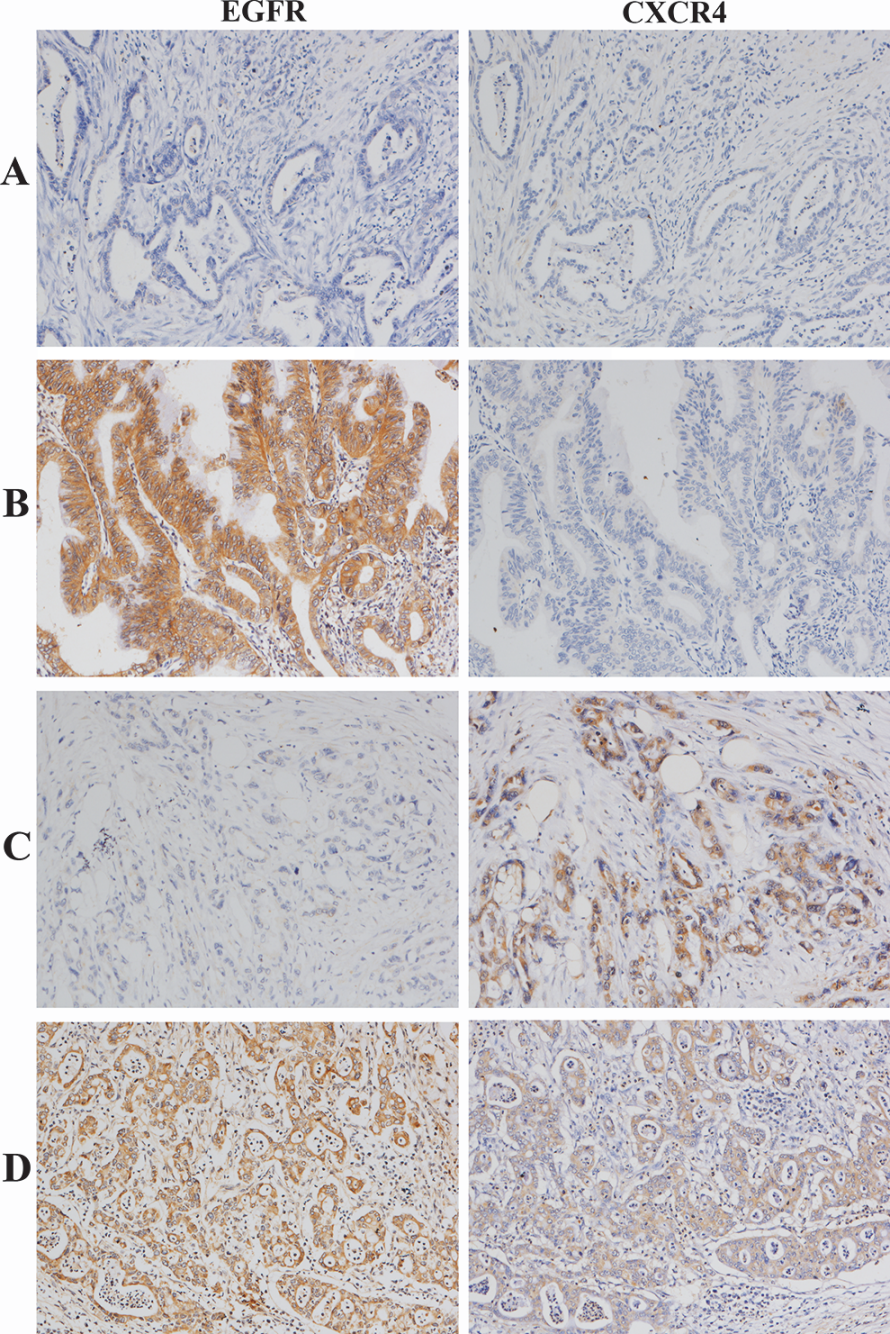
Representative PDAC cases with different EGFR/CXCR4 expression profiles: EGFR-/CXCR4− (A), EGFR+/CXCR4− (B), EGFR-/CXCR4+ (C), and EGFR+/CXCR4+ (D). Magnification x 100.

Immunohistochemical staining of EGFR and CXCR4 was observed to be both cytoplasmic and membranous in PDAC tissues. EGFR expression, CXCR4 expression and EGFR/CXCR4 coexpression were detected in 64 (48.9%), 68 (51.9%) and 33 (25.2%) of the 131 patients with PDAC, respectively. Representative PDAC tissues with different EGFR/CXCR4 expression profiles are shown in Fig. 1. The normal acinar and ductal cells of the peritumoral areas stained negatively or weakly for EGFR and CXCR4, whereas both EGFR and CXCR4 were moderately expressed in normal pancreatic islet cells. Furthermore, weak staining for CXCR4 was observed in a majority of the infiltrating inflammatory cells (S1 Fig.).

### Correlation between EGFR and CXCR4 expression

Among 131 PDAC patients, 31 were single-positive for EGFR, 35 were single-positive for CXCR4，33 were positive for both EGFR and CXCR4 expression, and 32 were negative for both EGFR and CXCR4 expression, respectively. Using the χ² test, there was no significant association between EGFR and CXCR4 expression (*P* = 0.938).

### Correlation of CXCR4 and EGFR expression with clinicopathologic characteristics in PDAC

A significant association between EGFR expression and poor differentiation was observed in our study (*P* = 0.031). CXCR4 expression was significantly associated with lymph node metastasis (*P* = 0.001), and EGFR/CXCR4 coexpression was significantly associated with lymph node metastasis (*P* = 0.026), TNM stage (*P* = 0.048), and poor tumor differentiation (*P* = 0.004) (Table 2).

**Table 2:** Correlation between EGFR/CXCR4 expression and clinicopathologic factors in PDAC.

Parameter	EGFR n (%)		P	CXCR4 n (%)		P	EGFR /CXCR4 n (%)		P
	Low	High		Low	High		Negative^a^	Positive^b^	
**Overall**	67 (51.1)	64 (48.9)		63 (48.1)	68 (51.9)		98 (74.8)	33 (25.2)	
**Age (years)**			0.163			0.223			0.842
<60	27 (40.3)	34 (53.1)		33 (52.4)	28 (41.2)		45(45.9)	16 (48.5)	
≥60	40 (59.7)	30 (46.9)		30 (47.6)	40 (58.8)		53 (54.1)	17 (51.5)	
**Gender**			0.599			1.000			0.689
Male	40(59.7)	35(54.7)		27 (42.9)	29 (42.6)		43 (43.9)	13 (39.4)	
Female	27 (40.3)	29 (45.3)		36 (57.1)	39 (57.4)		55 (56.1)	20 (60.6)	
**Tumor sites**			1.000			0.583			0.536
Head	43 (64.2)	42 (65.6)		39 (61.9)	46 (67.6)		62 (63.3)	23 (69.7)	
Body/tail	24 (35.8)	22 (34.4)		24 (38.1)	22 (32.4)		36 (36.7)	10 (30.3)	
**Tumor size**			0.220			0.117			0.068
T1–2	34(50.7)	25 (39.1)		33 (52.4)	26 (38.1)		49 (50.0)	10 (30.3)	
T3–4	33(49.3)	39 (60.9)		30 (47.6)	42 (61.8)		49 (50.0)	23 (69.7)	
**Lymph node metastasis**			0.862			**0.001**			**0.026**
No	33 (49.3)	30 (46.9)		40 (63.5)	23 (33.8)		53 (54.1)	10 (30.3)	
Yes	34 (50.7)	34 (53.1)		23 (36.5)	45(66.2)		45(45.9)	23 (69.7)	
**TNM stage**			0.161			0.083			**0.048**
I-II	63 (94.0)	56 (87.5)		60 (95.2)	59 (86.8)		92 (93.9)	27 (81.8)	
III	4 (6.0)	8 (12.5)		3 (4.8)	9 (13.2)		6 (6.1)	6 (18.2)	
**Resection margins**			1.000			0.417			1.000
Negative	51 (76.1)	49 (76.6)		46 (73.0)	54 (79.4)		75 (76.5)	25 (75.8)	
Positive	16 (23.9)	15 (23.4)		17 (27.0)	14 (20.6)		23 (23.5)	8 (24.2)	
**Tumor differentiation**			**0.031**			0.206			**0.004**
Well/moderate	49 (73.1)	35 (54.7)		44 (69.8)	40 (58.8)		70 (71.4)	14 (42.4)	
Poor	18 (26.9)	29 (45.3)		19 (30.2)	28 (41.2)		28 (28.6)	19 (57.6)	

^a^ Negative: single positive or both negative for EGFR and CXCR4.

^b^ Positive: EGFR /CXCR4 coexpression (double positive for EGFR and CXCR4).

### Prognostic significance of CXCR4 expression, EGFR expression and EGFR/CXCR4 coexpression in PDAC

The median follow-ups for DFS and OS were 33.4 months (range, 1–50 months) and 32.6 months (range, 2–53 months), respectively. The median DFS and OS were 11.6 months and 19.5 months, respectively.

**Figure 2:**
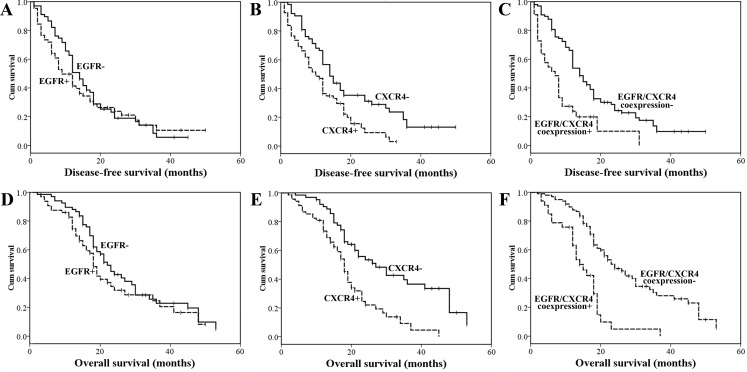
Kaplan-Meier analysis of DFS and OS in resected PDAC. DFS according to EGFR expression (*P* = 0.493) (A), CXCR4 expression (*P* = 0.003) (B) and EGFR/CXCR4 coexpression (*P*<0.001) (C). OS according to EGFR expression (*P* = 0.161) (D), CXCR4 expression (*P*<0.001) (E) and EGFR/CXCR4 coexpression (*P*<0.001) (F).

In the univariate survival analysis, lymph node metastasis, advanced TNM stage, positive resection margins, poor tumor differentiation, CXCR4 expression and EGFR/CXCR4 coexpression were significant prognostic factors for poor DFS and OS (Table 3, Fig. 2). Age≥60 years also predicted poor OS (Table 3). Patients with EGFR/CXCR4 coexpression had a significantly worse outcome than those without EGFR/CXCR4 coexpression (Fig. 2C and 2F, Table 3). To further explore the prognostic significance of the EGFR/CXCR4 interaction, the study population was divided into three groups: an EGFR/CXCR4 coexpression group (CXCR4+ and EGFR+), a single-positive group (either CXCR4+ or EGFR+) or a both-negative group (CXCR4− and EGFR-). Using the log-rank test, the EGFR/CXCR4 coexpression group was associated with a significantly worse prognosis than either the single-positive group (*P*<0.001 for both DFS and OS) or the both-negative group (*P* = 0.005 and <0.001 for DFS and OS, respectively), whereas no difference in survival was found between the single-positive group and the both-negative group (*P* = 0.720 and 0.545 for DFS and OS, respectively). If patients with EGFR/CXCR4 coexpression were excluded, neither EGFR nor CXCR4 protein expression significantly affected OS and DFS (data not shown).

**Table 3:** Univariate analysis for disease-free survival and overall survival.

Parameter	DFS			OS		
	HR	95% CI	P	HR	95% CI	P
**Age (years)**			0.153			0.029
<60	1			1		
≥60	1.31	0.89–1.94		1.58	1.03–2.42	
**Gender**			0.724			0.604
Male	1			1		
Female	0.94	0.64–1.37		0.61	0.59–1.36	
**Tumor sites**			0.732			0.580
Head	1			1		
Body/tail	1.07	0.72–1.59		1.13	0.73–1.76	
**Tumor size**			0.595			0.150
T1–2	1			1		
T3–4	1.11	0.76–1.62		1.35	0.89–2.05	
**Lymph node metastasis**			**0.020**			**0.006**
No	1			1		
Yes	1.55	1.05–2.28		1.77	1.16–2.71	
**TNM stage**			**<0.001**			**<0.001**
I-II	1			1		
III	4.29	2.28–8.10		3.32	1.73–6.37	
**Resection margins**			**0.022**			**<0.001**
Negative	1			1		
Positive	1.64	1.05–2.56		2.24	1.28–3.89	
**Tumor differentiation**			**0.000**			**<0.001**
Well/moderate	1			1		
Poor	2.07	1.38–3.09		2.41	1.57–3.69	
**EGFR expression**			0.493			0.161
Low	1			1		
High	1.14	0.78–1.66		1.34	0.88–2.03	
**CXCR4 expression**			**0.003**			**<0.001**
Low	1			1		
High	1.78	1.20–2.64		2.41	1.54–3.76	
**EGFR/CXCR4 coexpression**			**<0.001**	0.001		**<0.001**
Negative	1			1		
Positive	2.35	1.51–3.67		3.16	1.96–5.09	

The hazard ratio was estimated by Cox regression. EGFR/CXCR4 coexpression increased the hazard ratio for both recurrence and death and decreased the *P*-value compared with either EGFR or CXCR4 expression alone (Table 3). Additionally, by entering the three variables (EGFR expression, CXCR4 expression and EGFR/CXCR4 coexpression) into a multivariate model, the increase in the hazard ratio of EGFR/CXCR4 coexpression was significant (S1 Table).

Clinicopathologic factors at the 0.10 level in the univariate analysis were entered into a multivariate survival analysis model with EGFR/CXCR4 coexpression. Advanced TNM stage, positive resection margins, poor tumor differentiation and EGFR/CXCR4 coexpression were independent unfavorable prognostic factors for both DFS and OS. Moreover, age≥60 years and lymph node metastasis were also independent prognostic indicators for poor OS (Table 4).

**Table 4:** Multivariate analysis for disease-free survival and overall survival.

Parameter	HR	95% CI	p
***Disease-free survival: Cox regression model***			
**TNM stage**			
I-II	1		
III	3.86	2.01–7.43	<0.001
**Resection margins**			
Negative	1		
Positive	1.66	1.03–2.65	0.035
**Tumor differentiation**			
Well/moderate	1		
Poor	1.69	1.10–2.59	0.016
**EGFR/CXCR4 coexpression**			
Negative	1		
Positive	2.33	1.46–3.76	<0.001
			
***Overall survival: Cox regression model***			
**Age (years)**			
<60	1		
≥60	1.57	1.00–2.46	0.051
**Lymph node metastasis**			
No	1		
Yes	1.58	1.00–2.50	0.050
**TNM stage**			
I-II	1		
III	2.99	1.52–3.96	0.002
**Resection margins**			
Negative	1		
Positive	2.73	1.66–4.48	<0.001
**Tumor differentiation**			
Well/moderate	1		
Poor	2.50	1.58–3.96	<0.001
**EGFR/CXCR4 coexpression**			
Negative	1		
Positive	2.48	1.45–4.25	0.001

## Discussion

This is the first study to investigate the association between growth factor receptor EGFR expression and the expression of chemokine receptor CXCR4 and to assess the clinical and prognostic significance of their coexpression in PDAC. The present study showed that although there was no significant association between EGFR and CXCR4 expression, EGFR/CXCR4 coexpression was associated with poor tumor differentiation and predicted worse prognosis in PDAC. Moreover, EGFR/CXCR4 coexpression significantly increased the hazard ratio for both recurrence and death compared with the protein expression of either EGFR or CXCR4 alone and remained an independent prognostic factor for poor DFS and OS in PDAC according to a multivariate survival analysis.

Although it is well known that PDAC is a Kras-driven cancer, data from in vitro and murine in vivo tumor models reveal the critical role of EGFR in pancreatic tumorigenesis and development [19]. The EGFR-tyrosine kinase inhibitor (EGFR-TKI) erlotinib is the only targeted agent to have demonstrated a small but significant improved survival in advanced pancreatic cancer when combined with gemcitabine [20]. However, discordant results have been reported regarding EGFR overexpression in PDAC as a predictor of survival. According to previous immunohistochemical studies, EGFR was overexpressed in 7.7–100% of PDAC depending on the sample selection, antibody used, expression localization and the cut-off point that defines EGFR overexpression [21]. The majority of these studies have failed to demonstrate an association with prognosis [22]. In our study, EGFR overexpression was observed in 64 of 131 (48.9%) patients with resected PDAC and demonstrated limited clinical and prognostic significance. However, we observed a significant association between EGFR expression and poor differentiation. Consistent with our studies, Funel et al.[18] and Handra et al.[23] also found that tumor expression of EGFR was associated with tumor dedifferentiation but not with clinical outcome in PDAC. Although several other studies have demonstrated that EGFR expression was present in 30.4% of cases and was associated with lymph node and distant organ metastasis [24], [25], our study failed to demonstrate a significant relationship between EGFR expression and lymph node metastasis. As discussed above, our results indicated that although EGFR is a potential therapeutic target for pancreatic cancer, EGFR expression alone had limited clinical and prognostic significance in PDAC and the role of EGFR in PDAC carcinogenesis is complex, which is also supported by the fact that EGFR expression is not predictive of the erlotinib response in PDAC [21].

In recent years, investigators, including our group, have increasingly recognized the critical role of the tumor microenvironment in tumorigenesis and chemoresistance and the urgent need for the development of additional therapeutic strategies targeting tumor–stroma interactions [26], [27]. PDAC is clinically characterized by early locoregional spread and distant metastasis and is histologically characterized by the presence of abundant tumor stroma (desmoplasia). As a critical mediator of tumor–stroma interactions, CXCL12/CXCR4 axis plays an important role in the bidirectional tumor-stromal interaction, and therefore may be a promising prognostic marker and therapeutic target in PDAC [28], [29]. The results from both in vitro and in vivo studies have revealed that the stroma-induced CXCL12/CXCR4 axis is involved in pancreatic cancer metastasis through migration, invasion, and angiogenesis/lymphangiogenesis [30–32]. In support of these results, our study indicated that CXCR4 expression was significantly correlated with both lymph node metastasis and an unfavorable prognosis in resected PDAC and further supported the important role of tumor–stroma interactions in PDAC progression. Consistent with our report, Marechal et al.[10] and Bachet et al.[9] demonstrated that patients with CXCR4 overexpression have a worse outcome and a higher risk of distant metastatic recurrence in resected PDAC. Moreover, it has also been reported that CXCR4 expression is higher in pancreatic cancer cells derived from metastatic lesions compared with those derived from primary tumors [33]. Taken together with these results, our findings suggest that CXCR4 appears to be an important prognostic factor for metastasis and an attractive target in PDAC, particularly for antimetastatic therapy. It has been reported that CXCL12/CXCR4 expression was significantly correlated with microvascular density (MVD)/microlymphatic vessel density (MLVD), and thus might be associated with angiogenesis/lymphangiogenesis and organ-specific metastasis in pancreatic cancer [32]. However, Guo et al. [34] showed that there was no significant association between CXCR4 mRNA or protein expression and VEGF-C expression or lymph node metastasis in PDAC. Given the lack of MVD/MVLD analysis and CXCR4 mRNA expression analysis in our study, the correlation among CXCR4, MVD/MLVD and metastasis in PDAC needs further clarified.

Interactions between EGFR and CXCR4 have been described recently [11]-[13]. Although no significant correlation between the expressions of EGFR and CXCR4 was found in our study, we demonstrated for the first time that EGF/CXCR4 coexpression identified a subset of resected PDAC patients with more aggressive tumor characteristics and was associated with a significantly higher risk for both recurrence and death. Moreover, a multivariate analysis revealed that coexpression of EGFR and CXCR4 was an independent factor for poor survival in resected PDAC. Our results suggest that an important cross-talk between the two membrane receptors may also exist in PDAC, promoting an aggressive PDAC phenotype, thus indicating the potential for improved therapeutic efficacy by the simultaneous inhibition of EGFR and CXCR4. Given that the CXCL12/CXCR4 axis is considered to be a critical mediator of cancer–stroma interactions, our study also supports the important contributions of the tumor microenvironment to EGFR cross-talk with other signaling pathways in PDAC. However, given the limited sample size and the fact that the patients included in our series had relatively early-stage diseases, our results need further validation in multicentre studies with large cohorts of patients. Moreover, the mechanisms underlying EGFR and CXCR4 cooperation in PDAC have not been well elucidated and need to be further explored both in vitro and in vivo. One possible mechanism might be that they might interact with each other directly by regulating the activity of existing proteins rather than through regulating expression. This hypothesis is supported by a study indicating that CXCR4 could promote EGFR activation and subsequent downstream ERK activation in pancreatic cancer [35]. Another mechanism may be that EGFR and CXCR4 share common downstream signaling molecules. In support of this proposed mechanism, it has been reported that genes differentially regulated between high CXCR4 and low CXCR4 pathways are enriched with genes from the EGFR pathway [35].

In conclusion, we demonstrated for the first time that EGFR/CXCR4 coexpression identified a subset of PDAC patients with more aggressive clinicopathologic characteristics and a significantly worse prognosis. Our results suggested a possible important "cross-talk" between EGFR and CXCR4 intracellular pathways in PDAC. The mechanisms underlying the "cross-talk" need to be further explored. Our results also indicated that the simultaneous inhibition of these pathways might be an attractive therapeutic strategy for PDAC.

## Supporting Information

S1 Fig Representative examples of positive (A) and negative (B) EGFR staining and positive (C) and negative (D) CXCR4 staining in resected PDAC. Magnification x 200.
(TIF)

S1 Table Cox’s regression model for EGFR/CXCR4 coexpression. (DOC)
