# Supplementary figures and images for: Coexpression of EGFR and CXCR4 Predicts Poor Prognosis in Resected Pancreatic Ductal Adenocarcinoma
Source: PLoS One. 2015 Feb 13;10(2):e0116803. doi: 10.1371/journal.pone.0116803 (PMC4332630; doi:10.1371/journal.pone.0116803)

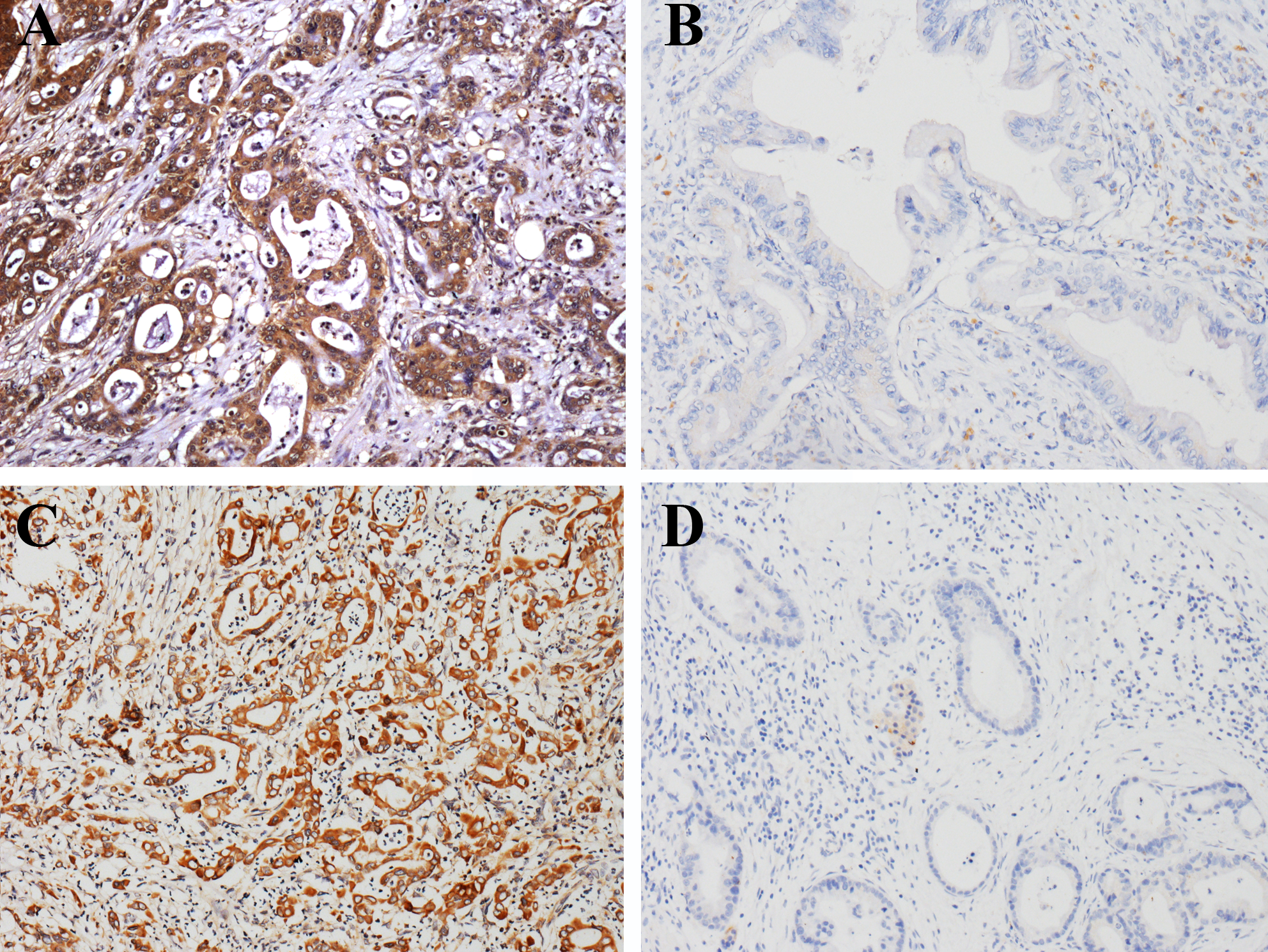

Supplement: S1 Fig — Magnification x 200. (TIF) [file pone.0116803.s001.tif]
