# Supplementary material for: Coexpression of EGFR and CXCR4 Predicts Poor Prognosis in Resected Pancreatic Ductal Adenocarcinoma
Source: PLoS One. 2015 Feb 13;10(2):e0116803. doi: 10.1371/journal.pone.0116803 (PMC4332630; doi:10.1371/journal.pone.0116803)
Supplement: S1 Table — (DOC) [file pone.0116803.s002.doc]

**Supporting Information**

Table S1. Cox’s regression model for EGFR/CXCR4 coexpression

|  | **DFS** | | |  | **OS** | | |
| --- | --- | --- | --- | --- | --- | --- | --- |
|  | **HR** | **95% CI** | ***p*** |  | **HR** | **95% CI** | ***p*** |
| **EGFR expression** |  |  | 0.408 |  |  |  | 0.813 |
| Low | 1 |  |  |  | 1 |  |  |
| High | 0.79 | 0.45-1.39 |  |  | 0.92 | 0.48-1.78 |  |
| **CXCR4 expression** |  |  | 0.453 |  |  |  | 0.089 |
| Low | 1 |  |  |  | 1 |  |  |
| High | 1.23 | 0.72-2.09 |  |  | 1.69 | 0.92-3.07 |  |
| **EGFR/CXCR4 coexpression** |  |  | **0.023** |  |  |  | **0.043** |
| Negative | 1 |  |  |  | 1 |  |  |
| Positive | 2.45 | 1.13-5.29 |  |  | 2.43 | 1.03-5.72 |  |
